# Supplementary material for: The interactome of intact mitochondria by cross-linking mass spectrometry provides evidence for coexisting respiratory supercomplexes
Source: Mol Cell Proteomics. 2017 Dec 8;17(2):216–32. doi: 10.1074/mcp.RA117.000470 (PMC5795388; doi:10.1074/mcp.RA117.000470)
Supplement: Supplemental Data [file supp_RA117.000470_133922_0_supp_23978_fzffwf.zip › spectra_annotation/mito_spectra_annotation/294-1-4-1-12-1.pdf]

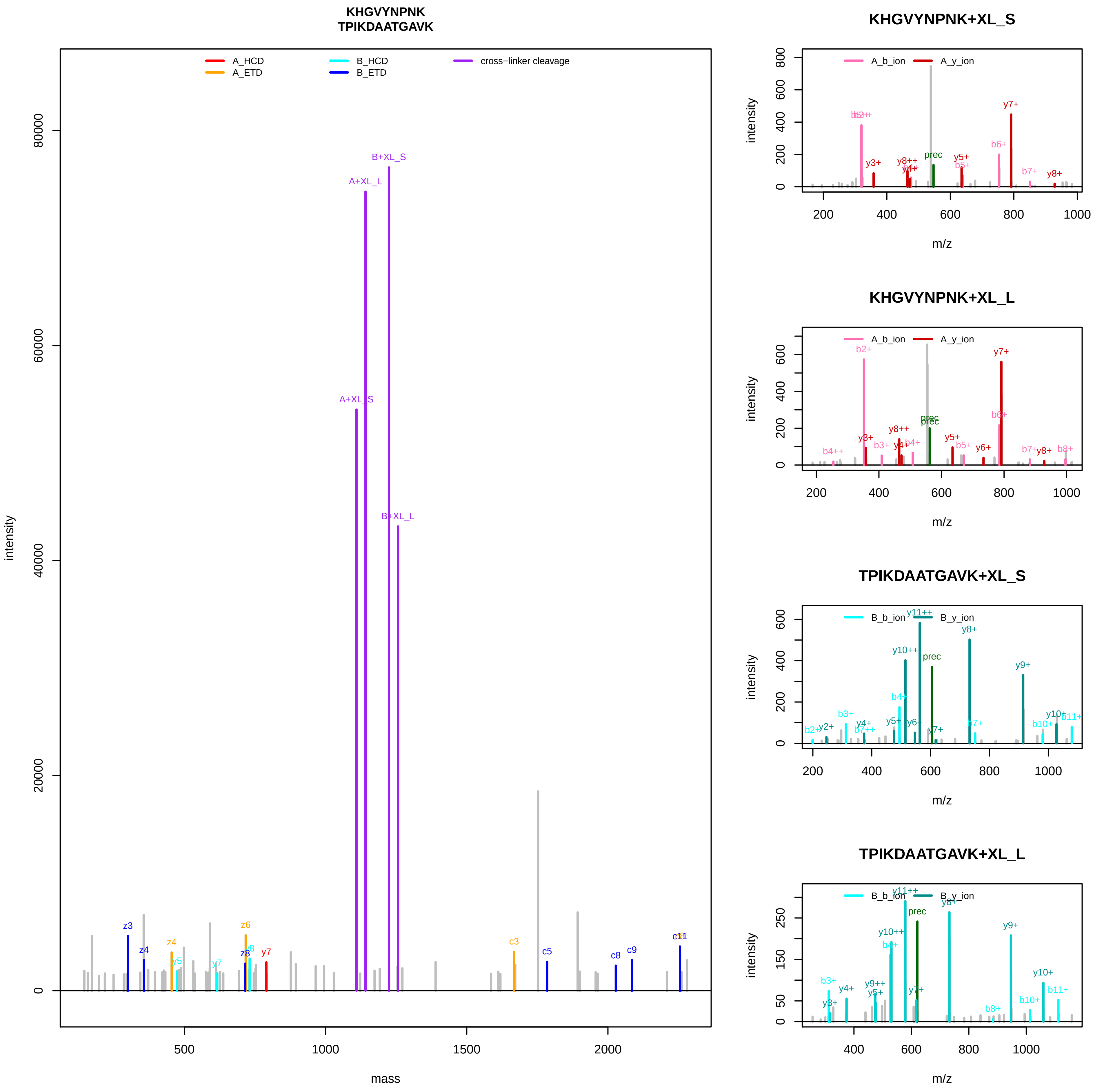
KHGVYNPNK
TPIKDAATGAVK
A_HCD
A_ETD
B_HCD
B_ETD
cross−linker cleavage
B+XL_S
A+XL_L
A+XL_S
B+XL_L
z3
z4
z4
y5
y7
z6
z8
y8
y7
c3
c5
c8
c9
c11
intensity
mass
0
20000
40000
60000
80000
500
1000
1500
2000
KHGVYNPNK+XL_S
A_b_ion
A_y_ion
KHGVYNPNK+XL_L
TPIKDAATGAVK+XL_S
B_b_ion
B_y_ion
TPIKDAATGAVK+XL_L
prec
m/z
intensity
